# A Mixture of *Ginkgo biloba L*. Leaf and *Hericium erinaceus (Bull.) Pers*. Fruit Extract Attenuates Scopolamine-Induced Memory Impairments in Mice

**DOI:** 10.1155/2022/9973678

**Published:** 2022-01-27

**Authors:** Seong Min Hong, Da Hye Yoon, Mi Kyeong Lee, Jae Kang Lee, Sun Yeou Kim

**Affiliations:** ^1^College of Pharmacy and Gachon Institute of Pharmaceutical Science, Gachon University, 191, Hambakmoe-ro, Yeonsu-gu, Incheon 21936, Republic of Korea; ^2^College of Pharmacy, Chungbuk National University, Cheongju 28160, Republic of Korea; ^3^CNGBio Co., Cheongju 28655, Republic of Korea

## Abstract

Alzheimer's disease (AD) is a neurodegenerative disease that is characterized by loss of memory and cognitive impairment *via* dysfunction of the cholinergic nervous system. In cholinergic dysfunction, it is well known that impaired cAMP response element-binding protein (CREB) and brain-derived neurotrophic factor (BDNF) signaling are major pathological markers and are some of the strategies for the development of AD therapy. Therefore, this study is aimed at evaluating whether a mixture comprising *Ginkgo biloba L*. leaf (GL) and *Hericium erinaceus (Bull.) Pers*. (HE) fruit extract (GH mixture) alleviated cognitive impairment induced in a scopolamine-induced model. It was discovered that GH reduced neuronal apoptosis and promoted neuronal survival by activating BDNF signaling in an *in vitro* assay. In addition, the GH (*p.o.* 240 mg/kg) oral administration group significantly restored the cognitive deficits of the scopolamine-induced mouse group (*i.p.* 1.2 mg/kg) in the behavior tests such as Y-maze and novel object recognition task (NORT) tests. This mixture also considerably enhanced cholinergic system function in the mouse brain. Furthermore, GH markedly upregulated the expressed levels of extracellular signal-regulated kinase (ERK), CREB, and BDNF protein levels. These results demonstrated that GH strongly exerted a neuroprotective effect on the scopolamine-induced mouse model, suggesting that an optimized mixture of GL and HE could be used as a good material for developing functional foods to aid in the prevention of neurodegenerative diseases, including AD.

## 1. Introduction

Alzheimer's disease (AD), which involves continuous memory and cognitive dysfunction, is an age-related neurodegenerative disease [1]. As mentioned in the World Health Organization (WHO) report, this disease is one of the rapidly increasing chronic diseases that is globally affecting approximately 30 million people over the age of 60 years each year [2]. Acetylcholine (ACh) released from the hippocampus and cortex plays a vital role in attention, memory, and learning [3]. However, the brain of AD patients has critically elevated levels of acetylcholinesterase (AChE), which is involved in the breakdown of ACh, resulting in a loss of memory and cognitive functions [4]. It is clear that dysfunction of cholinergic neurons is mainly involved in AD pathogenesis [5]. Hence, the development of AD therapy has mainly focused on the protective effect on the cholinergic system. Several drugs, as acetylcholinesterase inhibitors (*e.g.*, tacrine, rivatigmine, galantamine, and donepezil), have been approved for the treatment of AD, but they only help in keeping symptoms from worsening [6]. However, they have short half-lives and side effects such as hepatotoxicity and nausea [7]. Thus, it is needed to find alternative drugs to treat AD which show strong effects without any toxicity.

Many studies have shown that the scopolamine-induced AD model is useful for evaluating AD progression [8, 9]. Scopolamine, an ACh antagonist that leads to cholinergic dysfunction, is a tropane alkaloid that has been used for studying cognitive deficits in preclinical studies [10]. Numerous studies have already demonstrated that memory impairment in the scopolamine-induced mouse model is correlated with the production of oxidative stresses, such as through reactive oxygen species (ROS) from the brain *via* the dysfunction of antioxidant enzymes [11]. Moreover, it has been reported that scopolamine interferes with the neuronal protection mechanisms, including extracellular signal-regulated kinase (ERK) and cAMP response element-binding protein (CREB)/brain-derived neurotrophic factor (BDNF) mouse studies [12–14].

Meanwhile, cognitive enhancers, also known as nootropics, have been studied with respect to alleviating the suffering of AD patients [15]. Nootropics improve learning and memory through several mechanisms (*i.e.*, blockage of calcium channels, inhibition of AChE activities, enhancement of the levels of antioxidants, and synaptic and mitochondrial responses genes) [16]. Indeed, these agents also show neuroprotective effects *via* amyloid beta accumulation, synaptic dysfunctions, apoptosis, inflammation, and oxidative stress [15]. Many types of nootropics, which are drugs or herbal agents that improve memory function, have been reported [17]. The two kinds of nootropics are synthetic compounds (e.g., piracetam) and natural/herbal nootropics (*e.g.*, *Ginkgo biloba L*. leaf (GL) and *Hericium erinaceus (Bull.) Pers.* (HE)) [17–20]. In particular, natural nootropics act through various mechanisms in the brain [15]: (1) modulating neurotransmitter release, (2) improving neuronal function, (3) protecting against oxidative damage, and (4) serving as an energy booster. In addition, the application of nootropics for chronic treatment shows improvement in memory function *via* the regulation of ERK/CREB/BDNF pathways on the hippocampus. In view of the aforementioned points, nootropics from natural resources are recognized as alternative materials used to improve the cognitive impairment in AD.

Clinical studies with GL in dementia patients have pointed out that it slows down the decline in mental function and neuropsychiatric symptoms. In addition, GL showed efficacy and safety for treatment of cognitive impairment and dementia in clinical studies [21, 22]. HE revealed that it ameliorated BDNF levels and depressive behavior in chronically stressed animals, improved memory impairment, protected neurons from neurotoxicity (*e.g.*, beta-amyloid, hydrogen peroxide, and lipopolysaccharide), and restored hippocampal damage after pilocarpine-induced status epilepticus [19, 23–25]. The well-known edible nutrient nootropics, such as GL and HE, are cognitive enhancers. Nevertheless, it is reported that each such agent may not have any effect on memory dysfunction. This study was thus carried out to investigate the synergic effects of the improvement in cognitive function, both *in vitro* and *in vivo*.

## 2. Materials and Methods

### 2.1. Materials

Anti-Bax, anti-Bcl-2, anti-cleaved-caspase-3, anti-BDNF, anti-CREB, anti-phosphorylated CREB (pCREB), anti-ERK, anti-pERK, anti-protein kinase B (AKT), anti-pAKT, anti-glycogen synthase kinase 3 beta (GSK3*β*), anti-pGSK3*β*, and glyceraldehyde 3-phosphate dehydrogenase (GAPDH) were purchased from Cell Signaling Technology (Beverly, MA, USA). 3-(4,5-Dimethylthiazol-2-yl)-2,5-diphenyl-tetrazolium bromide (MTT), dimethyl sulfoxide (DMSO), 2′,7′-dichlorofluorescein diacetate (DCFH-DA), scopolamine, and donepezil hydrochloride were obtained from Sigma-Aldrich (St. Louis, MO, USA). The mitochondrial membrane potential assay kit (JC-10) and anti-doublecortin (DCX) were purchased from Abcam (Cambridge, MA, USA).

### 2.2. Preparation of GL and HE

The *G. biloba L*. leaf extract (lot number Kgb-191122, GL) containing 24% flavone, and <5 ppm ginkgolic acid was provided by SAS KPLC (Paris, France). *Hericium erinaceus (Bull.) Pers*. fruit was supplied by the CNGBio company (Cheongju, Korea). The dried fruit bodies of *H. erinaceus (Bull.) Pers*. were extracted two times with 70% EtOH for 2 h at 85°C and condensed using a rotary evaporator. Yield value of *H. erinaceus (Bull.) Pers*. (HE) extract was 43.32%. The GL and HE were prepared in DMSO and mixed in a 1 : 5 ratio (GH) for *in vitro* and *in vivo* tests.

### 2.3. High-Performance Liquid Chromatography (HPLC) Analysis of GH

GH, which was mixed in a ratio 1 : 5 of GL and HE, was analyzed using a Waters e2695 Separation Module (Waters Corp., Milford, MA, USA) with a 2998 Photodiode Array Detector. For the analysis of kaempferol, quercetin, isorhamnetin, and acacetin from GH, twenty milligrams of GH mixture and standard compounds (0.1, 1, and 10 *μ*g/mL) were dissolved in 50% acetonitrile/water. The mobile phase consisted of 0.4% phosphoric acid/water (solvent A) and acetonitrile (solvent B) with the following gradient: 0–10 min, 10% B; 10–40 min, 10–70% B; 40–44 min, 70–100% B; and 44–47 min, 0% B, with a flow rate of 1 mL/min [26]. For the analysis of hericene A and hericene D from GH, three milligrams of GH mixture and standard compounds (0.1, 1, and 10 *μ*g/mL) were dissolved in 30% ethyl acetate/methanol. For isocratic analyses, the mobile phase consisted of 97% methanol/3% water with a flow rate of 1 mL/min [27]. For the analysis of ginkgolide A and ginkgolide B, twenty milligrams of GH mixture and standard compounds (1, 10, and 100 *μ*g/mL) were dissolved in 50% methanol/water. For isocratic analyses, the mobile phase consisted of 72.5% water/17.5% water/10% isopropanol with a flow rate of 1 mL/min [28]. All analyses used a Kromasil C^18^ column (150 *mm* × 4.6 *mm*, 5 *μ*m). The column temperature was set at 30°C, and the PDA detector was set at 280-340 nm to acquire chromatograms.

### 2.4. Cell Culture

Human neuroblastoma SH-SY5Y cells were obtained from the Korea Cell Line Bank (Seoul, Korea) and maintained in Dulbecco's modified Eagle's medium (GIBCO/Invitrogen, Carlsbad, CA, USA) supplemented with 10% fetal bovine serum (GIBCO, Carlsbad, CA, USA) and 1% penicillin-streptomycin (GIBCO/Invitrogen, Carlsbad, CA, USA) at 37°C in a humidified atmosphere containing 5% CO_2_. SH-SY5Y cells were seeded in 96- and 6-well plates at densities of 4 × 10^4^ cells/well and 1 × 10^6^ cells/well, respectively.

### 2.5. Cell Viability

Cell viability was measured using a 2.4.3-(4,5-dimethylthiazol-2-yl)-2,5-diphenyltetrazolium bromide (MTT) assay according to Venkatesan et al. [29]. SH-SY5Y cells were seeded in 96-well plates and pretreated with various concentrations of GH (dissolved in PBS), hericene A, hericene D, ginkgolide A, ginkgolide B, isorhamnetin, acacetin, quercetin, and kaempferol. All standard components were dissolved in DMSO. After incubation for 1 h at 37°C, scopolamine was cotreated with GH for 24 h at 37°C. The reaction medium was removed, and 0.5 mg/mL of MTT solution was added. The treated cells were incubated at 37°C for 1 h. The medium was removed, and 200 *μ*L of DMSO was added. This assay was performed at 570 nm using a microplate reader (Bio-Rad, Hercules, CA, USA). Cell morphology was detected using a 10x objective (scale *bar* = 50 *μ*m) in the InCucyte ZOOM Live Cell Analysis System (Essen Instruments, Ann Arbor, MI, USA).

### 2.6. Measurement of Reactive Oxygen Species (ROS) Production

SH-SY5Y cells were seeded in 96-well plates and pretreated with various concentrations of GH (dissolved in PBS). After incubation for 1 h at 37°C, scopolamine was cotreated with GH for 1 h. Afterwards, 30 *μ*M DCFH-DA was added to the culture medium for 30 min at 37°C. The fluorescence of DCF, which is the oxidation product of DCFH-DA, was measured (*excitation* (*Ex*)/*emission* (*Em*) = 485/535 nm) using a VICTOR X3 plate reader (Perkin Elmer, Waltham, MA, USA) [30].

### 2.7. Measurement of Mitochondrial Membrane Potential

The mitochondrial membrane potential assay was carried out using a JC-10 kit (Abcam, CA, USA) [31]. SH-SY5Y cells were seeded in 96-well plates and pretreated with various concentrations of GH (dissolved in PBS). After incubation for 1 h at 37°C, scopolamine was cotreated with GH for 24 h at 37°C. After treatment, the cell mitochondria were stained using 50 *μ*L of JC-10 solution for 30 min at 37°C and kept away from light. After incubation, 50 *μ*L of buffer B solution was added into the JC-10 loading plate before reading the fluorescence intensity, and this was analyzed using a microplate reader (BioTek Instruments Inc., Winooski, VT, USA). The fluorescence intensities were measured at *Ex*/*Em* = 490/525 nm and 490/590 nm for ratio analysis.

### 2.8. Animal Study

**Figure 1:**
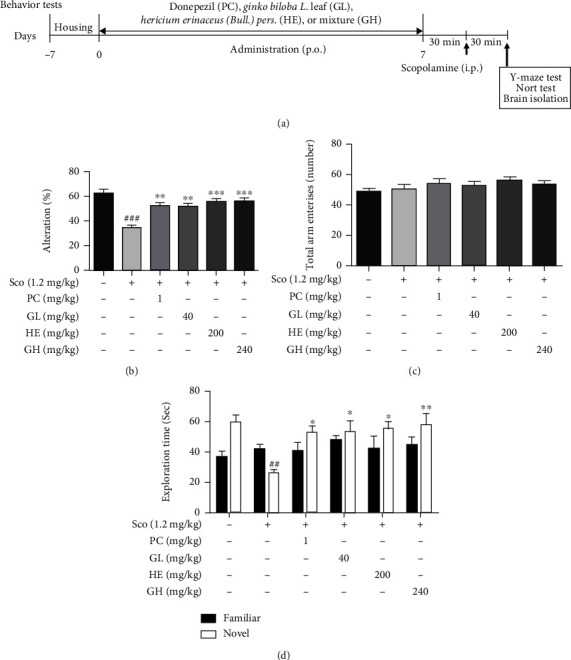
Effect of the mixture (GH) of *Ginkgo biloba L*. leaf (GL) and *Hericium erinaceus (Bull.) Pers.* (HE) fruit extracts on scopolamine- (Sco-) induced mouse model. (a) *In vivo* experiments were performed as shown in the schematic diagram. Each group was orally administered with GH (240 mg/kg), GL (40 mg/kg), HE (200 mg/kg), PC (1 mg/kg donepezil), or water. Before behavior tests (30 min), Sco was intraperitoneally injected for constructing the memory deficit model. (b, c) The Y-maze test was performed 30 min later. Spontaneous alternation percentage (b) and total numbers of arm entries (c) were recorded. (d) NORT test was performed 30 min later. The exploration times of the familiar and novel objects were recorded. Each group was orally administered with GH (240 mg/kg), GL (40 mg/kg), HE (200 mg/kg), PC (1 mg/kg donepezil), or water. Before behavior tests (30 min), Sco was intraperitoneally injected for constructing the memory deficit model. All behavior tests were analyzed by using video tracking software. Each behavior represents the mean ± SEM (*n* = 4–5). ^#^*P* < 0.05 and ^##^*P* < 0.01 vs. control group. ^∗∗^*P* < 0.01 and^∗∗∗^*P* < 0.001 vs. Sco-treated group.

Institute of Cancer Research (ICR, 6 weeks old, male) mice were obtained from Orient Bio Inc. (Gyeonggi-do, Korea). Each mouse was housed in ventilated cages under a specific pathogen-free environment (temperature, 23 ± 2°C; relative humidity, 55 ± 5%). Mice were fed an AIN-76A purified rodent diet (Central Laboratory Animal Inc., Seoul, Korea) and given water ad libitum. All mice (*n* = 60) were randomly divided into six groups (*n* = 10 per group): normal (10 mL/kg, per os (*p.o.*)), scopolamine (Sco, 1.2 mg/kg, intraperitoneal injection (*i.p.*)), donepezil (PC, 1 mg/kg, *p.o.*), *G. biloba L.* leaf (GL, 40 mg/kg, *p.o.*), *H. erinaceus (Bull.) Pers*. fruit (HE, 200 mg/kg, *p.o.*), and a mixture of GL and HE (GH, 240 mg/kg, *p.o.*). All samples were dissolved in 0.9% saline and administered for 1 week. Afterward, each group was induced with 1.2 mg/kg scopolamine (dissolved in 0.9% saline) 30 min before Y-maze and NORT tests on the first day. Body weight was measured once weekly from day 0 and did not change among groups (Figure 1S). The Y-maze (*n* = 5 per group) and novel objective recognition task (NORT (*n* = 5 per group)) were performed from day 7 to day 8 (see Figure 1(a)) [32, 33]. After behavior tests, all mice (*n* = 10 per group) were sacrificed and collected whole brain and then analyzed for histology (*n* = 3 per group) and biochemical assessments (*n* = 7 per group). This *in vivo* study was carried out according to the *Guide for the Care and Use of Laboratory Animals* and approved by the Institutional Animal Care and Use Committee of Gachon University, Korea (Approval No. GIACUC-R2019001-2). In addition, all behavior tests were analyzed by using SMART 3.0® video tracking software (Panlab, Barcelona, Spain).

### 2.9. Y-Maze Test

The Y-maze is a maze of three identical arms (60 × 15 × 12 cm^3^). Each arm was marked as A, B, and C. Each mouse was placed in the center of this tool, and the sequence (*e.g.*, ABC) and the numbers of arm entries were manually recorded through video analysis. In this behavior test, the actual alternation was defined as entries into arms on consecutive choices (i.e., ABC, BCA, or BAC but not ACA). This tool was cleaned using 70% ethanol. The percentage of spontaneous alternation performance (%) was calculated by using equation (1) [32]:

(1) $$\begin{array}{c} \frac{\left[\text{Actual alternations }\left(\text{total alternations}\right)\right]}{\left[\text{Possible alternation }\left(\text{total number of arm entries}–2\right)\right]}\times 100. \end{array}$$

### 2.10. Novel Object Recognition Task (NORT) Test

The NORT test was performed using an open field box (60 × 60 × 60 cm^3^). Each mouse was placed in the box with two different objects for 3 min on the 1^st^ day of training. The next day, mice were placed in the box again in which one of the identical objects had been replaced with a novel object. The exploration time of familiar and novel objects was recorded.

### 2.11. Acetylcholine (ACh) and Acetylcholinesterase (AChE) Activity Assay

After the behavioral tests, each mouse group was sacrificed, and the brain was collected and stored at -80°C. Before the ACh and AChE activity assay, whole brain (*n* = 7 per group) with PBS (*Ca.* 20-fold volumes) buffer was homogenized using an MP FastPrep-24 Instrument (MP Biomedicals Inc., SA, USA). The homogenates in PBS were centrifuged at 12,000 × *g* for 30 min at 4°C. The upper solution was used for ACh and AChE activity. The amount of brain lysate solution was calculated using the Bio-Rad Bradford kit (Bio-Rad).

ACh levels were analyzed using an EnzyChrom™ acetylcholine assay kit (BioAssay System, CA, USA). Briefly, 20 *μ*L of brain lysate solution was mixed with 80 *μ*L working reagent in a 96-well plate. The color intensity was detected using a microplate reader (Bio-Rad) at 570 nm from 0 min to 10 min, compared with that of the standard (ACh) [34].

AChE activity was analyzed using the QuantiChrom™ Acetylcholinesterase Assay Kit (BioAssay System). The AChE assay was conducted in a 96-well plate, and 10 *μ*L of brain lysate solution was mixed with a 190 *μ*L working reagent in a 96-well plate. The intensities of samples were detected using a microplate reader (Bio-Rad) at 412 nm from 0 min to 10 min [34]. AChE activity of each group was calculated as the percentage, compared to the control group.

### 2.12. Western Blotting Assay

SH-SY5Y cells or brain tissues (*n* = 7 per group) were lysed in PRO-PREP™ buffer (iNtRON Biotechnology, Seongnam, Korea) to obtain proteins. The protein amount from each sample was calculated using the Bio-Rad Bradford kit (Bio-Rad). An equal amount (30 *μ*g) of protein was loaded and separated using 10–12% SDS-PAGE gel and transferred onto a nitrocellulose membrane (Millipore Corp., MA, USA). Blocking was performed with 5% skimmed milk for 2 h, and the blots were incubated for 1 d with primary antibodies against Bax, Bcl-2, cleaved-caspase-3, BDNF, CREB, pCREB, ERK, pERK, AKT, pAKT, GSK3*β*, pGSK3*β*, and GAPDH (dilution 1 : 1000). The blots were then incubated with secondary antibodies (dilution, 1 : 2000). Protein bands were analyzed using the Chemi DocXRS^+^imaging system (Bio-Rad).

### 2.13. Histopathological Analysis

We obtained whole mouse brain by carrying out perfusion for blood removal according to a previous study [27]. Brain tissues (*n* = 3 per group) were collected from all groups and then fixed in 10% formalin. Fixed tissues were processed to obtain 4 *μ*m paraffin-embedded sections. Each section was stained with hematoxylin and eosin (H&E) and analyzed at 4–40x magnification using a microscope (Olympus, Tokyo, Japan). The optical density of H&E-stained tissue sections in the dentate gyrus (DG), cornu ammonis 1 (CA1), and cornu ammonis 2/3 (CA2/3) regions was analyzed using the ImageJ software (Bethesda, MD, USA).

### 2.14. Immunohistochemistry Analysis

The obtained 4 *μ*m paraffin-embedded sections (*n* = 3 per group) were deparaffinized using xylene and rehydrated with EtOH (100%, 90%, 80%, and 70%), followed by treatment with an endogenous peroxidase blocker, and finally washed with PBS. These sections were incubated with the primary antibody, BDNF (dilution 1 : 500), at 4°C. After washing in PBS, each section was incubated with a biotinylated anti-goat and anti-rabbit IgG (dilution 1 : 200) for 1 h and then with avidin-biotin horseradish peroxidase complex (Vector Laboratories, CA, USA). The optical density of BDNF immunoreactivity in the CA1 and CA2/3 regions was analyzed using ImageJ software. The images were photographed at 4–40x magnification using a microscope (Olympus).

### 2.15. Statistical Analysis

In this study, all data were analyzed using GraphPad Prism 5 software (CA, USA) and represented as *means* ± *standard* *error* of the *mean* (SEM). All results were analyzed using one-way analysis of variance (ANOVA), followed by Tukey-Kramer's *t*-test for post hoc analysis. Differences with a *P* value less than 0.05 were considered statistically significant.

## 3. Results

### 3.1. GH Improves Neuroprotective Effects on Scopolamine-Induced Cytotoxicity in SH-SY5Y Cells

**Figure 2:**
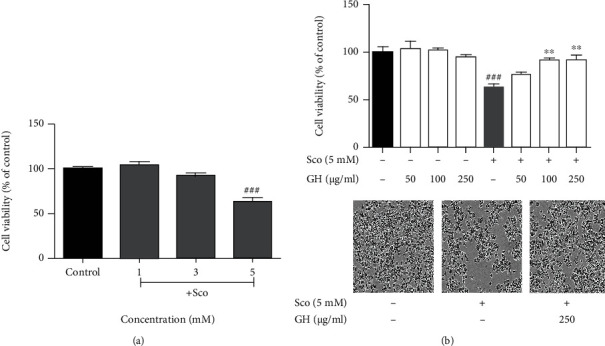
Effect of the mixture (GH) of *Ginkgo biloba L*. leaf (GL) and *Hericium erinaceus (Bull.) Pers*. (HE) fruit extracts on scopolamine- (Sco-) induced cytotoxicity in human neuroblastoma (SH-SY5Y) cells. (a) Cells were treated with Sco at 1, 3, and 5 mM concentrations. Cell viability was measured by MTT assay. (b) Cells were pretreated with 50, 100, and 500 *μ*g/mL GH for 1 h and then cotreated with 5 mM Sco cotreated for 24 h. Cell viability was measured by MTT assay. Morphological images of cells were observed by using a microscope (scale bar = 50 *μ*m). Data represent the mean ± SEM (*n* = 3). ^###^*P* < 0.001 vs. control group. ^∗∗^*P* < 0.01 vs. Sco-treated group.

Scopolamine showed cytotoxicity in neuronal cells with cholinergic dysfunction [10]. For studying the cytotoxicity effect of scopolamine in SH-SY5Y cells, the SH-SY5Y cells were treated with 1, 3, or 5 mM scopolamine for 1 day. As shown in Figure 2(a), treatment with 5 mM scopolamine significantly decreased cell viability (63.33 ± 3.96%, ^###^*P* < 0.001) compared to that of the control group. Therefore, further tests were performed using 5 mM scopolamine to investigate the neuroprotective effects of GH on scopolamine-induced cytotoxicity. In addition, GH was mixed with GL and HE in a 1 : 5 ratio, since the cytotoxicity of GL and HE on scopolamine-induced SH-SY5Y cells was significantly suppressed at 50 *μ*g/mL (GL, 22.02 ± 5.14%,  ^∗^*P* < 0.05) and 250 *μ*g/mL (HE, 24.82 ± 2.28%,  ^∗∗^*P* < 0.001), respectively. SH-SY5Y cells were pretreated with 50, 100, and 250 *μ*g/mL GH for 1 h and then cotreated with 5 mM scopolamine for 24 h. In the MTT assay, GH significantly increased cell viability compared with that in the negative (scopolamine only) group (Figure 2(b)). Moreover, the MTT assay showed that GH did not exert any cytotoxicity. As shown in Figure 2(b), the treatment of scopolamine progressively changed the cellular morphology such as shrinkage of cells and decreased density of cells. Meanwhile, pretreatment with 250 *μ*g/mL GH prevented the observed situation. These results suggest that GH prevented scopolamine-induced cytotoxicity.

### 3.2. Phytochemical Analysis of GH by Using HPLC System

**Figure 3:**
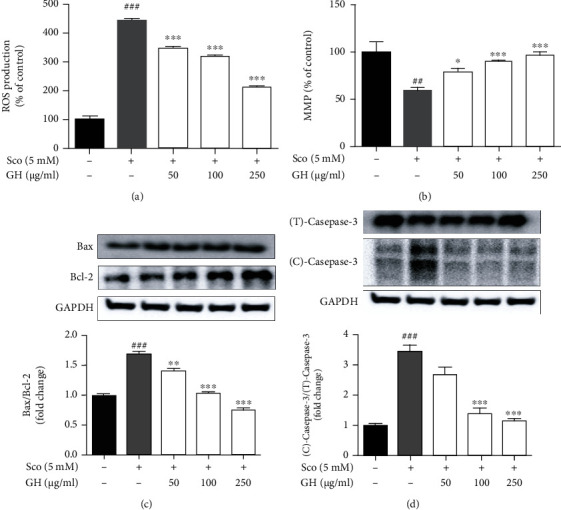
Effect of the mixture (GH) of *Ginkgo biloba L*. leaf (GL) and *Hericium erinaceus (Bull.) Pers.* (HE) fruit extracts on scopolamine- (Sco-) induced ROS production, mitochondrial dysfunction, and apoptosis pathways in human neuroblastoma (SH-SY5Y) cells. (a) ROS production was measured using the DCF-DA staining assay. (b) Mitochondrial membrane potential was measured for evaluating mitochondrial dysfunction using the JC-10 assay kit. (c, d) Western blotting assay of Bax, Bcl-2, cleaved- (C-) caspase-3, and total- (T-) caspase-3 was performed, with GAPDH as a loading control. Quantification was calculated using densitometric analysis using Bio-Rad Quantity software. Data represent the mean ± SEM (*n* = 3). ^##^*P* < 0.01 and ^###^*P* < 0.001 vs. control group. ^∗∗^*P* < 0.01 and^∗∗∗^*P* < 0.001 vs. Sco-treated group.

**Figure 4:**
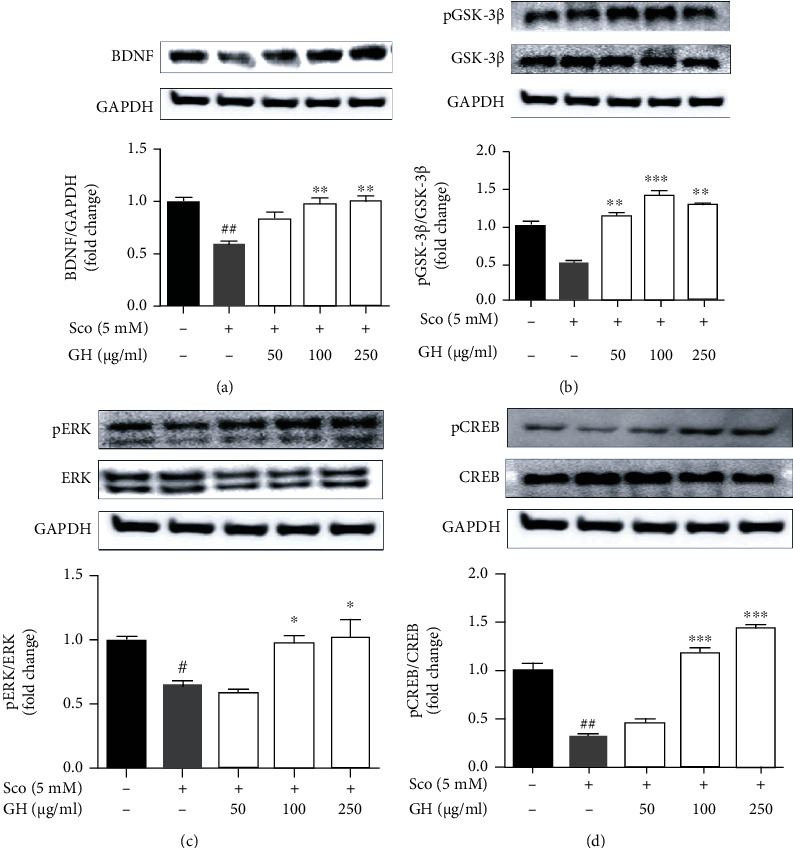
Neuronal protective effect of the mixture (GH) of *Ginkgo biloba L*. leaf (GL) and *Hericium erinaceus (Bull.) Pers*. (HE) fruit extracts on scopolamine- (Sco-) induced SH-SY5Y neuroblastoma cells. (a–d) Western blotting assay of BDNF (a), pGSK3*β*/GSK3*β* (b), pERK/ERK (c), and pCREB/CREB (d) was carried out. GAPDH was used as a loading control. Quantification was calculated using densitometric analysis using Bio-Rad Quantity software. Data represent the mean ± SEM (*n* = 3). ^#^*P* < 0.05 and ^##^*P* < 0.01 vs. control group. ^∗^*P* < 0.05,  ^∗∗^*P* < 0.01, and^∗∗∗^*P* < 0.001 vs. Sco-treated group.

**Figure 5:**
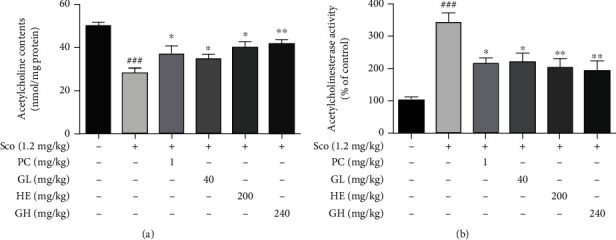
Effect of the mixture (GH) of *Ginkgo biloba L.* leaf (GL) and *Hericium erinaceus (Bull.) Pers*. (HE) fruit extracts on acetylcholine (ACh) level and acetylcholinesterase (AChE) activity in the brain. (a) ACh level in the brain lysates. (b) AChE activity in the brain lysates. Data represent the mean ± SEM (*n* = 7). ^#^*P* < 0.05 and ^##^*P* < 0.01 vs. control group. ^∗∗^*P* < 0.01 and^∗∗∗^*P* < 0.001 vs. Sco-treated group.

In our study, we analyzed several components from GH mixture extract. As shown in Table 1 and Figure 3S-5S, GH mixture mainly involved ginkgolide A (12.632 ± 0.605 mg/g), ginkgolide B (4.846 ± 0.538 mg/g), quercetin (7.693 ± 0.741 mg/g), and hericene A (1.669 ± 0.030 mg/g). In addition, other components as kaempferol, hericene D, isorhamnetin, and acacetin were detected for 0.591 ± 0.029 mg/g, 0.578 ± 0.005 mg/g, 0.026 ± 0.002 mg/g, and 0.0030 ± 0.0001 mg/g, respectively.

### 3.3. GH Prevents the Scopolamine-Induced ROS, Mitochondrial Dysfunction, and Apoptosis Pathways in SH-SY5Y Cells

To evaluate whether GH-treated neuroprotective effects were regulated via the suppression of apoptosis pathways, we first measured ROS production in SH-SY5Y cells. The scopolamine-treated group (445.60 ± 6.29%,  ^###^*P* < 0.001) showed a significant increase in ROS production compared with that in the control group, as shown in Figure 3(a). In addition, we measured the protection of mitochondrial dysfunction as a major hallmark of apoptosis in SH-SY5Y cells (Figure 3(b)). The scopolamine-treated group (60.20 ± 1.18%,  ^##^*P* < 0.01) demonstrated a significant loss in mitochondrial membrane potential, whereas GH-treated groups significantly recovered the loss of mitochondrial membrane potential in a dose-dependent manner. In particular, the scopolamine-treated group (^###^*P* < 0.001) showed increased levels of apoptosis-related proteins as the Bax/Bcl-2 ratio and cleaved-caspase-3, while GH-treated groups showed a significant decrease in the protein levels (Figures 4(c) and 4(d)). These results indicated that GH has a neuroprotective effect by reducing ROS production and mitochondrial dysfunction and suppressing the apoptosis pathways.

### 3.4. GH Increases the Levels of BDNF and Activates GSK3*β*, ERK, and CREB Signaling Pathways in Scopolamine-Induced SH-SY5Y Cells

To investigate the neuronal protection pathways in scopolamine-induced SH-SY5Y cells, we confirmed the signal transduction pathways (*e.g.*, cell survival, growth, and proliferation), including BDNF, GSK3*β*, CREB, and ERK signaling (Figure 4). Our data revealed that scopolamine significantly decreased BDNF, pGSK3*β*, pCREB, and pERK, whereas GH treatment inhibited the effects of scopolamine on the levels of pGSK3*β*, pCREB, and pERK. It was reported that BDNF, GSK3*β*, CREB, and ERK signaling play vital roles in the process of memory, learning, and modulation of cholinergic function, suggesting that GH has a neuroprotective effect via regulation of these signaling pathways [12].

### 3.5. GH Improves Scopolamine-Induced Memory Impairment in Behavior Tests

For elucidating the neuroprotective effects of GH on cognitive memory function, we carried out two behavior tests on the scopolamine-induced mouse model: Y-maze and NORT tests (Figure 1(a)). The mouse model used was six-week-old ICR males and was orally administered GL (40 mg/kg, *p.o.*), HE (200 mg/kg, *p.o.*), or GL+HE (GH, 240 mg/kg, *p.o.*) for 7 days before scopolamine injection (1.2 mg/kg, *i.p.*). In particular, the administration dose of GH was also mixed with GL and HE in a 1 : 5 ratio as shown *in vitro* data, and the administration dose of GL was referred from previous findings [35, 36]. In addition, the positive control was used donepezil (1 mg/kg, *p.o.*), which is used for treating AD.

In the Y-maze test, each group was allowed to explore the Y-maze for 8 min, and the spontaneous alternation percentage and total number of arm entries were recorded. The spontaneous alternation percentage in the scopolamine (1.2 mg/kg, *i.p.*)-treated mice group was critically decreased (35.09 ± 6.27%,  ^##^*P* < 0.01) than that of the normal group (62.38 ± 3.47%) (Figure 1(b)). The decreased spontaneous alternation by scopolamine was significantly improved by treatment with 240 mg/kg GH (56.36 ± 2.35%,  ^∗∗^*P* < 0.01). Interestingly, it was observed that the spontaneous alternation by GH treatment was higher than that in the other groups (40 mg/kg GL, 52.09 ± 2.42%; 200 mg/kg HE, 55.64 ± 2.11%; and 1 mg/kg PC (donepezil), 53.02 ± 2.55%), implying that there was a synergistic effect of GH administration on enhancing memory function. In addition, the difference in the number of total entries did not impact the results of the Y-maze (Figure 1(c)).

The role of GH on cognitive memory was also assessed using the NORT test. The scopolamine group (26.48 ± 1.97 sec,  ^##^*P* < 0.01) showed a significant decrease in exploration in novel places, compared with that in the normal group (59.86 ± 4.95 sec). Meanwhile, the administration of 240 mg/kg GH (57.78 ± 9.54 sec,  ^∗∗^*P* < 0.01) significantly increased the exploration time at novel places compared to that in the 1 mg/kg PC group (53.47 ± 3.84 sec,  ^∗^*P* < 0.05). Indeed, the exploring time was significantly higher for the 240 mg/kg GH-, 200 mg/kg HE (55.50 ± 4.49,  ^∗^*P* < 0.05)-, 1 mg/kg PC (53.47 ± 3.84 sec,  ^∗^*P* < 0.05)-, and 40 mg/kg GL (53.80 ± 8.74 sec,  ^∗^*P* < 0.05)-treated groups compared with that in the scopolamine group, respectively (Figure 1(d)).

### 3.6. GH Restores the ACh Level and AChE Activity in the Scopolamine-Induced Mouse Brain

The ACh level and AChE activity in the mouse brain lysates were measured using respective assay kits [34]. As shown in Figure 5(a), our data revealed that the ACh level in the brain was markedly decreased in the scopolamine-induced group (27.98 ± 2.16 nmol/mg protein,  ^###^*P* < 0.01) than that in normal mice (49.92 ± 1.81 nmol/mg protein). The ACh content of the GH group (41.55 ± 1.83 nmol/mg protein) was higher than that of the scopolamine group. Meanwhile, AChE activity was significantly higher in the scopolamine group (340.68 ± 32.91%,  ^###^*P* < 0.001) than that in the normal group (100.00 ± 11.57%), but the GH group (191.37 ± 31.63%,  ^∗∗^*P* < 0.01) showed that AChE activity was markedly decreased as similar to that in the PC group (214.17 ± 18.80%,  ^∗^*P* < 0.05) (Figure 5(b)).

### 3.7. GH Modulates the BDNF/ERK/CREB Pathways in the Mouse Brain

**Figure 6:**
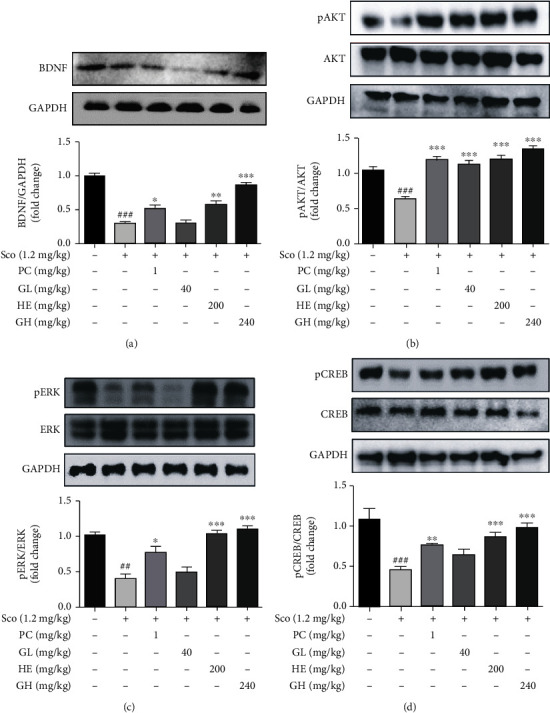
Effect of the mixture (GH) of *Ginkgo biloba L*. leaf (GL) and *Hericium erinaceus (Bull.) Pers*. (HE) fruit extracts on the expressed levels of BDNF, pAKT, pERK, and pCREB in the brain. Whole brain from randomly selected mice in each group was analyzed for western blotting assay. (a–d) Western blotting assay of BDNF (a), pAKT/AKT (b), pERK/ERK (c), and pCREB/CREB (d) was carried out. GAPDH was used as a loading control. Quantification was performed using densitometric analysis with Bio-Rad Quantity software. Data represent the mean ± SEM (*n* = 7).^#^*P* < 0.05 and^##^*P* < 0.01 vs. control group. ^∗∗^*P* < 0.01 and^∗∗∗^*P* < 0.001 vs. Sco-treated group.

To evaluate the related molecular mechanisms underlying the neuroprotective effects of GH, the protein levels of BDNF, pAKT, pERK, and pCREB in the mouse brain lysates were confirmed (Figure 6). The scopolamine group (^###^*P* < 0.001) significantly decreased the expression level of BDNF in the mouse brain, but GH administration (^∗∗∗^*P* < 0.01) significantly reversed it (Figure 6(a)). In addition, the expression levels of pAKT in the GH group (^∗∗∗^*P* < 0.001) were higher than that in the other groups (Figure 6(b)). Moreover, GH administration significantly increased the scopolamine-induced pERK (^∗∗∗^*P* < 0.001) and pCREB (^∗∗∗^*P* < 0.001) in the mouse brain (Figures 6(c) and 6(d)). This indicates that GH improves the scopolamine-induced decrease in the expression level of BDNF as well as ERK/CREB phosphorylation in the mouse brain, which may be involved in alleviating memory impairment.

### 3.8. GH Ameliorates the Pathological Changes of Hippocampus in Mouse Brain Tissue

**Figure 7:**
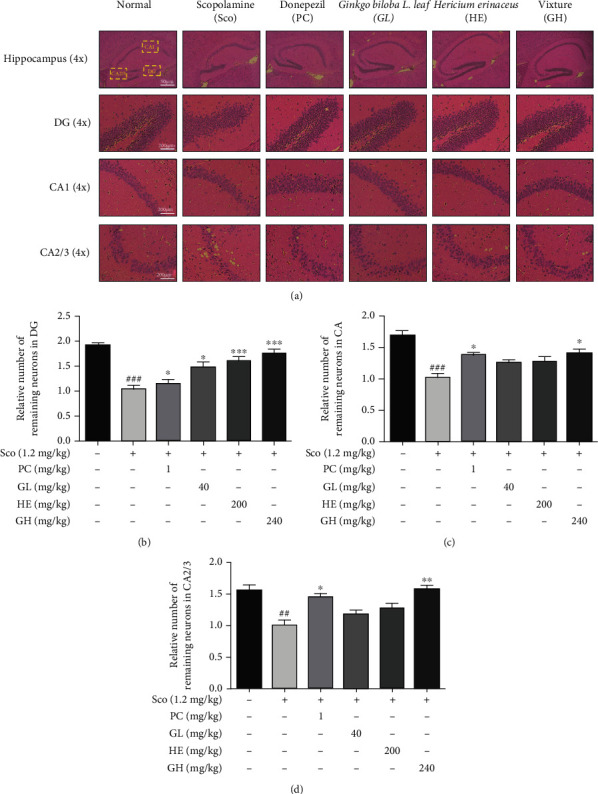
Effect of the mixture (GH) of *Ginkgo biloba L.* leaf (GL) and *Hericium erinaceus (Bull.) Pers.* (HE) fruit extracts on H&E staining assay. (a) The morphological changes of DG, CA1, and CA2/3 region from hippocampus. (b–d) The number of live neurons in each region. The area of DG, CA1, and CA2/3 regions was measured and quantified by using Micron (EVOS, v2.0) digital imaging software. Data represent the mean ± SEM (*n* = 3). ^##^*P* < 0.01 and^###^*P* < 0.001 vs. control group. ^∗^*P* < 0.05,  ^∗∗^*P* < 0.01, and^∗∗∗^*P* < 0.001 vs. Sco-treated group.

Histopathological changes in the mouse hippocampus were observed by the H&E staining assay (Figure 7). Neuronal damage was investigated in the dentate gyrus (DG), cornu ammonis (CA) 1, and CA2/3 region of the hippocampus in terms of the number of viable neurons, by using a microscope (40x magnification). The brain sections of normal mice displayed the normal structure of the hippocampus, such as the DG, CA1, and CA2/3 areas. In the scopolamine group, pyramidal cells of the stratum pyramidalis in the DG, CA1, and CA2/3 regions showed that scopolamine (^###^*P* < 0.001) caused significant damage in each region of the hippocampus, compared with those of the normal group. However, GH administration revealed a marked reversal of scopolamine-induced cell damage, similar to that by donepezil administration, indicating its neuroprotective potential.

### 3.9. GH Promotes the BDNF Expression in the Mouse Hippocampus

**Figure 8:**
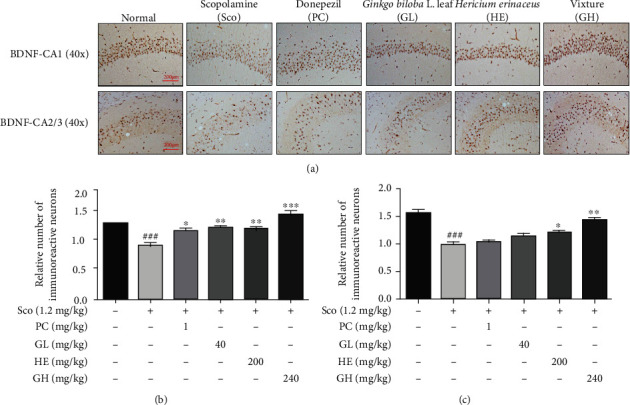
Effect of the mixture (GH) of *Ginkgo biloba L.* leaf (GL) and *Hericium erinaceus (Bull.) Pers.* (HE) fruit extracts on BDNF immunostaining assay. (a) The morphological changes of CA1 and CA2/3 region from hippocampus. (b, c) The number of live neurons in each region. The area of CA1 and CA2/3 regions was measured and quantified by using Micron (EVOS, v2.0) digital imaging software. Data represent the mean ± SEM (*n* = 3). ^###^*P* < 0.001 vs. control group. ^∗^*P* < 0.05,  ^∗∗^*P* < 0.01, and^∗∗∗^*P* < 0.001 vs. Sco-treated group.

BDNF, as a biomarker for synaptogenesis and synaptic plasticity, engages memory function by promoting neuronal cell survival and differentiation in hippocampal neurons [37]. In the present study, we examined whether GH mediates synapse formation in the scopolamine-induced hippocampus in mice by detecting the number of immunoreactive neurons in the CA1 and CA2/3 regions. The relative number of BDNF-reactive neurons in the CA1 and CA2/3 regions was significantly higher in both donepezil (^∗^*P* < 0.05) and GH-treated (^∗∗∗^*P* < 0.001) groups in comparison to that in the normal group (Figure 8). Our data suggest that the administration of GH may be able to induce the expression of BDNF, which is attributed to memory enhancement.

### 3.10. Phytochemicals from GH Improves Neuroprotective Effects on Scopolamine-Induced Cytotoxicity in SH-SY5Y Cells

**Figure 9:**
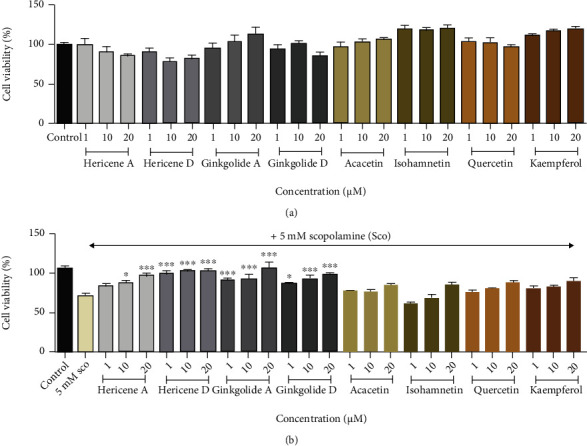
Effect of several components from the mixture (GH) of *Ginkgo biloba L.* leaf (GL) and *Hericium erinaceus (Bull.) Pers.* (HE) fruit extracts on scopolamine- (Sco-) induced SH-SY5Y neuroblastoma cells. (a) Cells were treated with hericene A, hericene D, ginkgolide A, ginkgolide B, isorhamnetin, acacetin, quercetin, and kaempferol at 1, 10, and 20 *μ*M concentrations. Cell viability was measured by MTT assay. (b) Cells were pretreated with 1, 10, and 20 *μ*M hericene A, hericene D, ginkgolide A, ginkgolide B, isorhamnetin, acacetin, quercetin, and kaempferol for 1 h and then cotreated with 5 mM Sco cotreated for 24 h. Cell viability was measured by MTT assay. Data represent the mean ± SEM (*n* = 3). ^###^*P* < 0.001 vs. control group. ^∗^*P* < 0.05,  ^∗∗^*P* < 0.01, and^∗∗∗^*P* < 0.001 vs. Sco-treated group.

As shown in Figure 9, the neuroprotective effects of phytochemicals as hericene A, hericene D, ginkgolide A, ginkgolide B, isorhamnetin, acacetin, quercetin, and kaempferol identified from GH were measured on scopolamine-induced cytotoxicity in SH-SY5Y cells. All phytochemicals prevented the cytotoxicity induced by scopolamine in a dose-dependent manner, and no cytotoxicity was detected. In particular, ginkgolide A, ginkgolide B, hericene A, and hericene D mainly prevented the scopolamine-induced cytotoxicity at 20 *μ*M.

## 4. Discussion

In this study, GH showed the effect of neuron protection on scopolamine-induced SH-SY5Y cells and improved scopolamine-induced cognitive impairments in a mouse model *via* behavioral tests including Y-maze and NORT. Moreover, we measured the neuroprotective mechanism of GH *via in vitro* and *in vivo* models. Interestingly, GH increased BDNF expression by activating CREB/ERK phosphorylation and downregulating ROS and AChE activities.

Previous studies on the pharmacological effects of GL and HE extracts in GH mixture may partly explain our data [17, 29]. GL ameliorates learning and memory impairments in animal studies and affects neurotransmitter levels, neuroplasticity, and suppression of the brain edema in clinical studies [15, 18]. HE extract showed great potential in improving the intellectual function of patients with cognitive impairment or against neurodegenerative diseases such as dementia and AD in the previous studies [29–31]. However, there is no report on a mixture of involved GL and HE showing a synergic effect on the scopolamine-induced mouse model. Thus, GL and HE were extracted from raw plant materials and arbitrarily mixed with a ratio of 1 : 5, since GL and HE significantly prevented scopolamine-induced cytotoxicity at 50 and 250 *μ*g/mL concentrations, in scopolamine-induced cytotoxicity, respectively (see Figure 2S). Furthermore, the mixture showed a synergic effect in comparison with each extract of GL or HE extracts on the scopolamine-induced mouse model (Figures 1, 5*–*8).

Scopolamine has been widely used to investigate a model of amnesia in a preclinical study that contributes to increased oxidative stress with impairment of memory and cognitive functions [14]. GH significantly downregulated scopolamine-induced cytotoxicity (Figures 2(b) and 2(c)) and ROS (Figure 3(a) and Figure 6S) and showed greater protection against neuronal cell death (Figure 3). Furthermore, GH suppressed the scopolamine-induced dephosphorylation of GSK3*β*, ERK, and CREB, which play vital roles in cell proliferation and survival signaling pathways (Figure 4). It was revealed that the inhibition of GSK3*β* by regulating pAKT reduces neuronal loss by suppressing apoptotic agents [38]. The activation of BDNF, ERK, and CREB signaling is related to neuronal cell survival and proliferation [12, 14]. It seems that the neuronal protection effect of GH on the scopolamine-induced model may be mainly modulated by BDNF and then activated by CREB and ERK phosphorylation.

In our *in vivo* results by using behavioral tests (Y-maze test, NORT test), it was found that GH improved scopolamine-induced memory impairments. These scopolamine-induced mouse models are widely used to analyze short-term memory [32]. Donepezil, an AChE inhibitor, is commonly used for treating AD, and it has also been shown to positively affect episodic memory function [39]. Thus, donepezil was used as a positive control in our designed *in vivo* model. In this study, GH displayed a similar or superior activity to that of donepezil (Figure 1), indicating that GH may be involved in cognitive enhancement.

BDNF affects synaptogenesis and synaptic plasticity in the central nervous system (CNS). Neurotrophic signaling involving ERK, CERB, and AKT is stimulated by the activation of BDNF, which contributes to memory and thinking. Indeed, GH considerably increased the expression levels of pERK, pCREB, and BDNF in the mouse brain (Figure 6), similar to that in *in vitro* data. Moreover, GL or HE could modulate BDNF/CREB/ERK in the brain and attenuate cognitive deficits in an amnesia mouse model. This indicates that the activation of the BDNF-induced signaling cascade by treating GL and/or HE could have neuroprotective effects on a scopolamine-induced mouse model (Figure 6). Furthermore, GH showed highly immunoreactive neurons by BDNF in the CA1 and CA2/3 hippocampal regions, indicating that GH treatment can increase synaptogenesis that is associated with boosting memory function (Figure 8).

Furthermore, the number of neurons observed by staining the doublecortin (DCX) was measured in the DG region of the hippocampus. DCX, a cytoskeletal protein, is expressed through neuronal cells and is widely used as a biomarker for neurogenesis [40]. Scopolamine refers to a decrease in DCX-immunoreactive cells in the DG and damage of dendritic development of the new immature neurons [41]. The decreased levels of stained DCX in the scopolamine-induced group were clearly observed. In the GH group, the number of immunoreactive neurons in the DG region was also improved in the scopolamine-induced mouse model, suggesting that GH engages in immature neuron differentiation in the hippocampus (Figure 7S).

Previous studies revealed that anti-AChE agents enhance the levels of ACh in the synaptic cleft and restore memory deficits [37]. Therefore, we also evaluated the effect of GH on the levels of ACh and AChE in a scopolamine-induced mouse model. Scopolamine increased AChE activity (0.61 ± 0.09 units/mg protein) and reduced ACh levels (12.15 ± 3.32 nmol/mg protein). Meanwhile, the administration of GH significantly reversed each level (AChE activity, 0.26 ± 0.03 units/mg protein; ACh level, 29.96 ± 8.62 nmol/mg protein) in the mouse brain (Figure 5). GH might be an efficient potential therapeutic source for preventing cholinergic dysfunction associated with AD. Also, we found that HE can strongly increase the content of ACh, while the activity of AChE became lower than that by GL treatment. This finding was in agreement with a few previous studies, which demonstrated that the AChE inhibitory activity of HE extracts involving hericenone and hericene components showed a mild effect [39, 40].

The major constituents of GH were identified as ginkgolide A (12.632 ± 0.605 mg/g), ginkgolide B (4.846 ± 0.538 mg/g), quercetin (7.693 ± 0.741 mg/g), and hericene A (1.669 ± 0.030 mg/g) by the HPLC system (Table 1 and Figures 3S-5S). Indeed, these compounds significantly prevented cytotoxicity on scopolamine-induced SH-SY5Y cells (Figure 9). Terpene trilactone such as ginkgolide A and ginkgolide B purified from GH has anti-platelet-activating factor, antiapoptotic, antioxidative, neurotrophic, and neuroimmunomodulatory effects [42]. In our previous study, hericene A as a HE metabolite reported that it increased the expression of nerve growth factor (NGF), BDNF, and synaptophysin, which promote neuronal survival and neurite outgrowth [43]. Hericene D isolated from HE also showed the potent neuroprotective effect against endoplasmic reticulum (ER) stress-dependent cell death. In addition, kaempferol, quercetin, isorhamnetin, and acacetin from GL, as flavone compounds, revealed that these compounds prevented effects of brain injury and neuroinflammation and provided neuronal protection [44, 45]. Therefore, these compounds may be the active components of GH involved in neuroprotection *in vitro* and *in vivo*. The underlying mechanism of the combined effects of those for neuronal protection and synaptic plasticity in the scopolamine-induced model requires further investigation.

## 5. Conclusions

In conclusion, our results showed that mixture of *G. biloba L.* leaf (GL) and *H. erinaceus (Bull.) Pers.* (HE) fruit extracts protected neuronal cells against scopolamine-induced neuronal dysfunction. Furthermore, GH ameliorated scopolamine-induced memory impairments as observed through Y-maze and NORT behavior tests. Moreover, GH prevented cognitive impairment *via B*DNF/ERK/CREB pathways in a scopolamine-induced mouse model. In addition, this mixture suppressed AChE activity in the mouse brain. All evidence suggests that GH could be a useful functional nutrient for preventing cognitive impairment. Additionally, further research should be carried out on the synergic effects of GH mixture on neuronal protection in a dose-dependent manner.

## Figures and Tables

**Table 1:** Contents of hericene A, hericene D, ginkgolide A, ginkgolide B, isorhamnetin, acacetin, quercetin, and kaempferol compounds by HPLC analysis in the presence of GH mixture.

Samples	Content (mg/g)							
	Hericene A	Hericene D	Ginkgolide A	Ginkgolide B	Isorhamnetin	Acacetin	Quercetin	Kaempferol
GH mixture	1.669 ± 0.030	0.578 ± 0.005	12.632 ± 0.605	4.846 ± 0.538	0.026 ± 0.002	0.0030 ± 0.0001	7.693 ± 0.741	0.591 ± 0.029

Data represent the mean ± SEM (*n* = 3).

## Data Availability

The data used to support the findings of this study are included within the supplementary information files.

## Abbreviations

ACh: Acetylcholine
AChE: Acetylcholinesterase
AD: Alzheimer's disease
AKT: Protein kinase B
BDNF: Brain-derived neurotrophic factor
CA1: Cornu ammonis 1
CA2/3: Cornu ammonis 2/3
CNS: Central nervous system
CREB: cAMP response element-binding protein
DCFH-DA: 2′, 7′-Dichlorofluorescein diacetate
DCX: Doublecortin
DG: Dentate gyrus
DMSO: Dimethyl sulfoxide
ERK: Extracellular signal-regulated kinase
GAPDH: Glyceraldehyde 3-phosphate dehydrogenase
GH mixture: A mixture of GL and HE extract
GL: *Ginkgo biloba L.* leaf extract
GSK3*β*: Glycogen synthase kinase 3 beta
HE: *Hericium erinaceus (Bull.) Pers.*
MTT: 3-(4,5-Dimethylthiazol-2-yl)-2,5-diphenyl-tetrazolium bromide
NGF: Nerve growth factor
NORT: Novel object recognition task
ROS: Reactive oxygen species.

## Supplementary Materials

Supplementary Materials Figure 1S: body weight transition after each sample administration for 10 days. Each group was orally administered GH (240 mg/kg, mixture (GH) of *Ginkgo biloba L.* leaf (GL) and *Hericium erinaceus (Bull.) Pers.* (HE) fruit extracts), GL (40 mg/kg, *Ginkgo biloba L.* leaf), HE (200 mg/kg, *Hericium erinaceus (Bull.) Pers* fruit), PC (1 mg/kg, donepezil), or water. After 30 min, Sco induced by intraperitoneal injection for making the memory deficit model. Data represent the mean ± SEM (*n* = 10). Figure 2S: effect of *Ginkgo biloba L.* leaf (GL) and *Hericium erinaceus (Bull.) Pers.* (HE) fruit extracts on scopolamine- (Sco-) induced cytotoxicity in SH-SY5Y neuroblastoma cells. (A, B) Cells were treated with GL or HE at 10, 25, 50, 100, and 250 *μ*g/mL concentrations. Cell viability was measured by the MTT assay. (C, D) Cells were treated with GL or HE at 10, 25, 50, 100, and 250 *μ*g/mL for 1 h, and then, 5 mM Sco was cotreated for 24 h. Cell viability was measured by the MTT assay. Data represent the mean ± SEM (*n* = 3). ^###^*P* < 0.001 vs. control group. ^∗^*P* < 0.05 and^∗∗^*P* < 0.01 vs. Sco-treated group. Figure 3S: chromatograms of standard compounds (quercetin, kaempferol, isorhamnetin, and acacetin, A) and GH mixture extract (B). Amounts of each compound were analyzed using HPLC with 0.1% trifluoroacetic acid (solvent A) and methanol/0.1% trifluoroacetic acid (solvent B) gradient system at UV 280 nm. Figure 4S: chromatograms of standard compounds (hericene A and hericene D) and GH mixture extract (B). Amounts of each compound were analyzed using HPLC with 0.1% trifluoroacetic acid (solvent A) and methanol/0.1% trifluoroacetic acid (solvent B) isocratic system at UV 294 nm. Figure 5S: chromatograms of standard compounds (ginkgolide A and ginkgolide D) and GH mixture extract (B). Amounts of each compound were analyzed using HPLC with 72.5% water/17.5% water/10% isopropanol isocratic system at UV 340 nm. Figure 6S: effect of the mixture (GH) of *Ginkgo biloba L.* leaf (GL) and *Hericium erinaceus (Bull.) Pers.* (HE) fruit extracts on scopolamine- (Sco-) induced ROS production. ROS production was measured using the DCF-DA staining assay. Cells were treated with GH (dissolved in PBS) at 50, 100, and 250 *μ*g/mL for 1 h, and then, 5 mM Sco was cotreated for 1 h. Green fluorescence (ROS generation) from 2′,7′-DCF-DA was examined by the JuLI live-cell imaging system. Scale bar indicates 500 *μ*m. Figure 7S: effect of the mixture (GH) of *Ginkgo biloba L.* leaf (GL) and *Hericium erinaceus (Bull.) Pers.* (HE) fruit extracts on doublecortin (DCX) immunostaining assay. The morphological changes of dentate gyrus (DG) region from hippocampus.
