# Supplementary material for: A Mixture of Ginkgo biloba L. Leaf and Hericium erinaceus (Bull.) Pers. Fruit Extract Attenuates Scopolamine-Induced Memory Impairments in Mice
Source: Oxid Med Cell Longev. 2022 Jan 27;2022:9973678. doi: 10.1155/2022/9973678 (PMC8813274; doi:10.1155/2022/9973678)
Supplement: Supplementary Materials — Figure 1S: body weight transition after each sample administration for 10 days. Each group was orally administered GH (240 mg/kg, mixture (GH) of Ginkgo biloba L. leaf (GL) and Hericium erinaceus (Bull.) Pers. (HE) fruit extracts), GL (40 mg/kg, Ginkgo biloba L. leaf), HE (200 mg/kg, Hericium erinaceus (Bull.) Pers fruit), PC (1 mg/kg, donepezil), or water. After 30 min, Sco induced by intraperitoneal injection for making the memory deficit model. Data represent the mean ± SEM (n = 10). Figure 2S: effect of Ginkgo biloba L. leaf (GL) and Hericium erinaceus (Bull.) Pers. (HE) fruit extracts on scopolamine- (Sco-) induced cytotoxicity in SH-SY5Y neuroblastoma cells. (A, B) Cells were treated with GL or HE at 10, 25, 50, 100, and 250 μg/mL concentrations. Cell viability was measured by the MTT assay. (C, D) Cells were treated with GL or HE at 10, 25, 50, 100, and 250 μg/mL for 1 h, and then, 5 mM Sco was cotreated for 24 h. Cell viability was measured by the MTT assay. Data represent the mean ± SEM (n = 3). ###P < 0.001 vs. control group. ∗P < 0.05 and∗∗P < 0.01 vs. Sco-treated group. Figure 3S: chromatograms of standard compounds (quercetin, kaempferol, isorhamnetin, and acacetin, A) and GH mixture extract (B). Amounts of each compound were analyzed using HPLC with 0.1% trifluoroacetic acid (solvent A) and methanol/0.1% trifluoroacetic acid (solvent B) gradient system at UV 280 nm. Figure 4S: chromatograms of standard compounds (hericene A and hericene D) and GH mixture extract (B). Amounts of each compound were analyzed using HPLC with 0.1% trifluoroacetic acid (solvent A) and methanol/0.1% trifluoroacetic acid (solvent B) isocratic system at UV 294 nm. Figure 5S: chromatograms of standard compounds (ginkgolide A and ginkgolide D) and GH mixture extract (B). Amounts of each compound were analyzed using HPLC with 72.5% water/17.5% water/10% isopropanol isocratic system at UV 340 nm. Figure 6S: effect of the mixture (GH) of Ginkgo biloba L. leaf (GL) and Hericium erina [file 9973678.f1.docx]

<Supplementary files>

**Figure 1S. Body weight transition after each sample administration for 10 days**. Each group was orally administered GH (240 mg/kg, mixture (GH) of *Ginkgo biloba L.* leaf (GL) and *Hericium erinaceus (Bull.) Pers.* fruit (HE) extracts), GL (40 mg/kg, *Ginkgo biloba L.* leaf), HE (200 mg/kg, *Hericium erinaceus (Bull.) Pers* fruit), PC (1 mg/kg, donepezil), or water. After 30 min, Sco induce by intraperitoneal injection for making memory deficit model. Data represent mean ± SEM (n = 10).

**
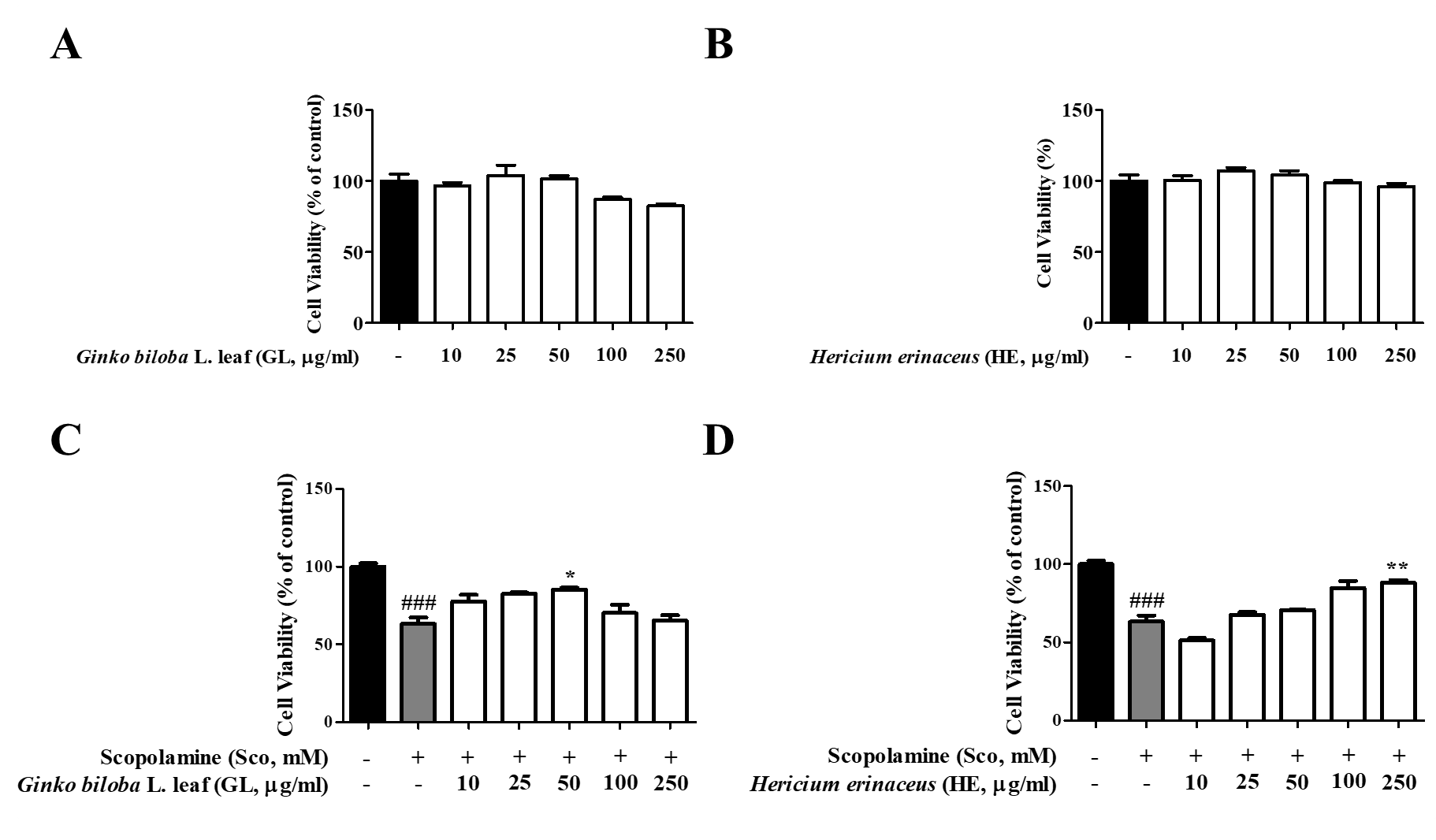
**

**Figure 2S. Effect of *Ginkgo biloba L.* leaf (GL) and *Hericium erinaceus (Bull.) Pers.* fruit (HE) extracts on scopolamine (Sco)-induced cytotoxicity in SH-SY5Y neuroblastoma cells.** (A, B) Cells were treated with GL or HE at 10, 25, 50, 100, and 250 μg/ml concentrations. Cell viability was measured by MTT assay. (C, D) Cells were treated with GL or HE at 10, 25, 50, 100, and 250 μg/ml for 1 h, and then 5 mM Sco was co-treated for 24 h. Cell viability was measured by MTT assay. Data represent mean ± SEM (n = 3). ^###^*P* < 0.001 vs. control group. ^*^*P* < 0.05 and ^**^*P* < 0.01 vs. Sco-treated group.


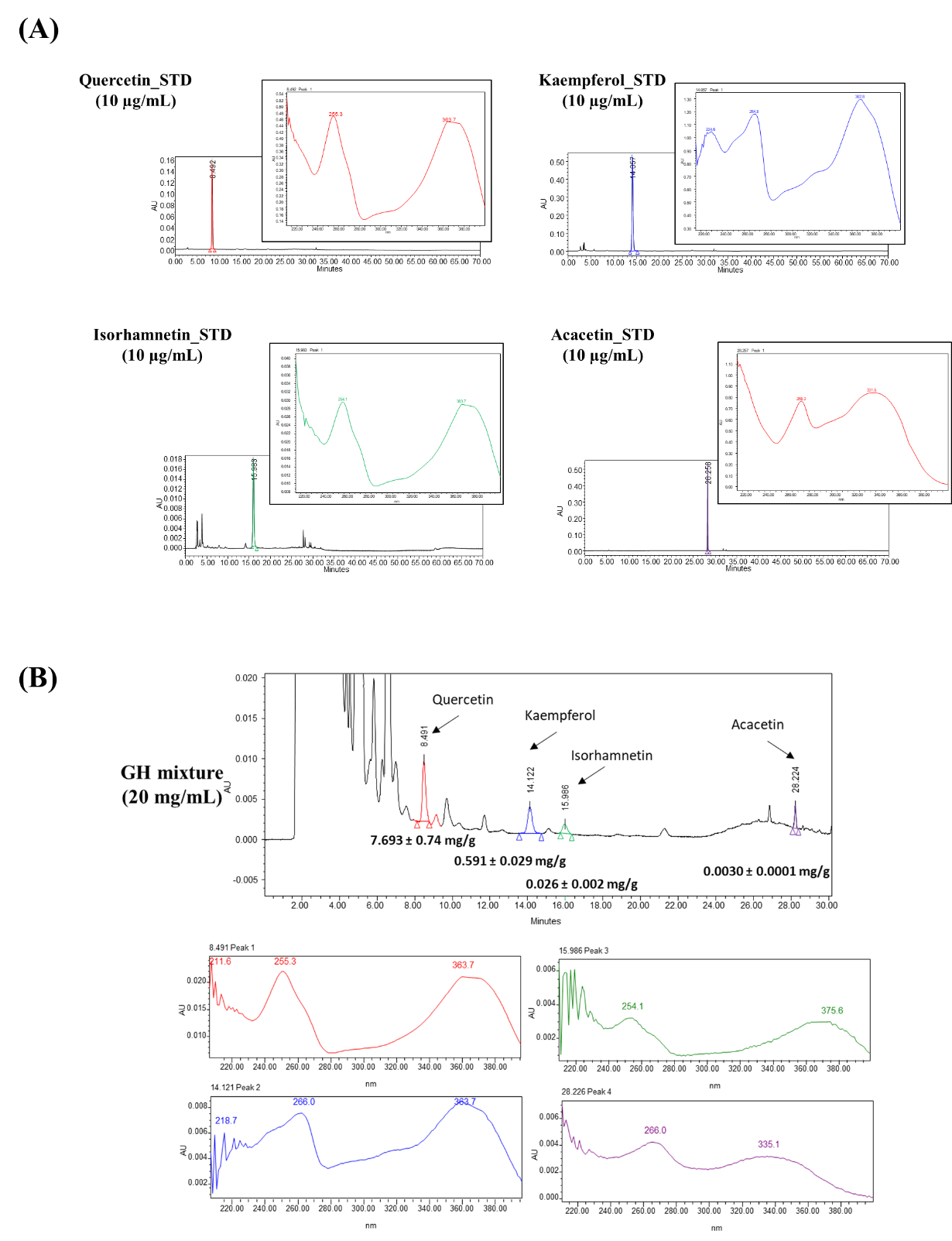


**Figure 3S. Chromatograms of standard compounds (quercetin, kaempferol, isorhamnetin, and acacetin, A) and GH mixture extract (B).** Amounts of each compound was analyzed using HPLC with 0.1% trifluoroacetic acid (solvent A) and methanol/0.1% trifluoroacetic acid (solvent B) gradient system at UV 280 nm.


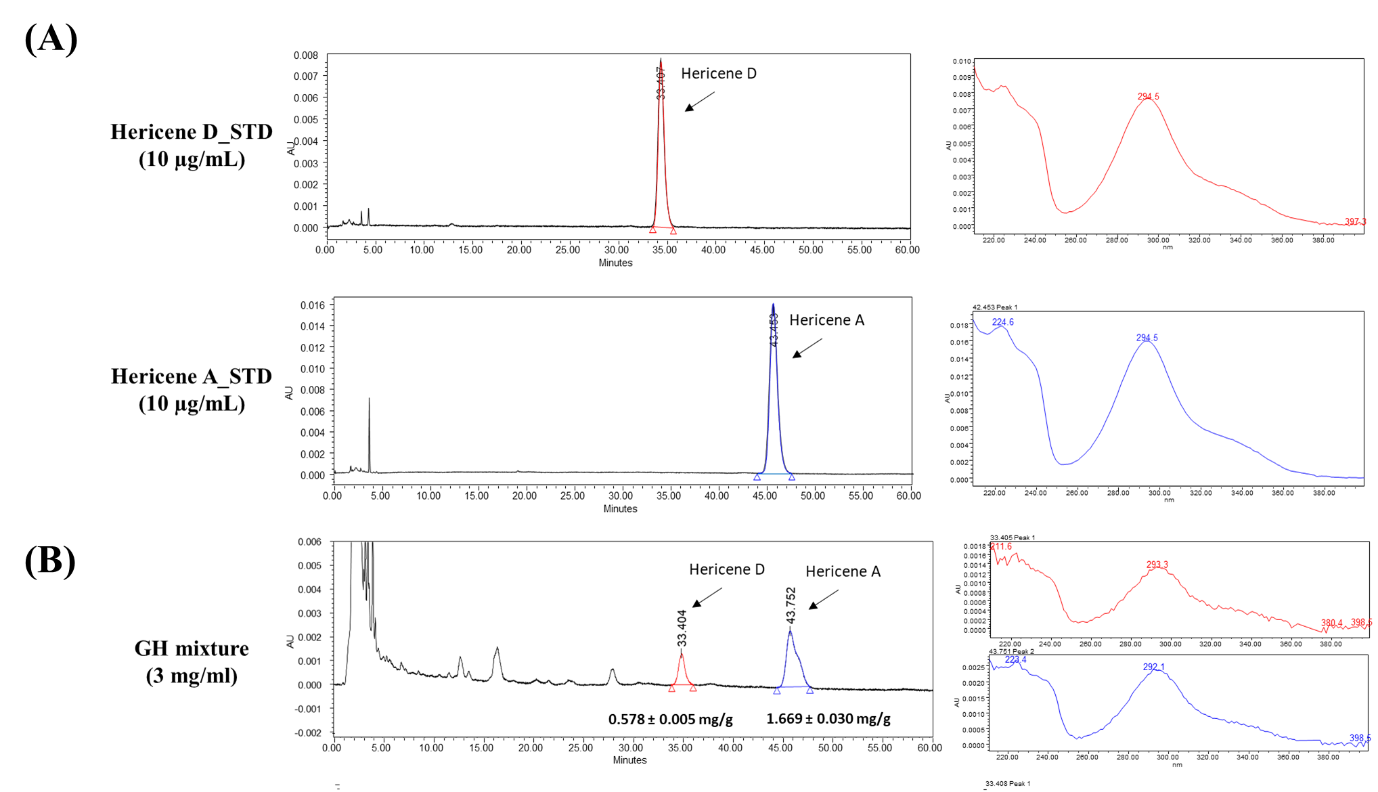


**Figure 4S. Chromatograms of standard compounds (hericene A and hericene D) and GH mixture extract (B).** Amounts of each compound was analyzed using HPLC with 0.1% trifluoroacetic acid (solvent A) and methanol/0.1% trifluoroacetic acid (solvent B) isocratic system at UV 294 nm.


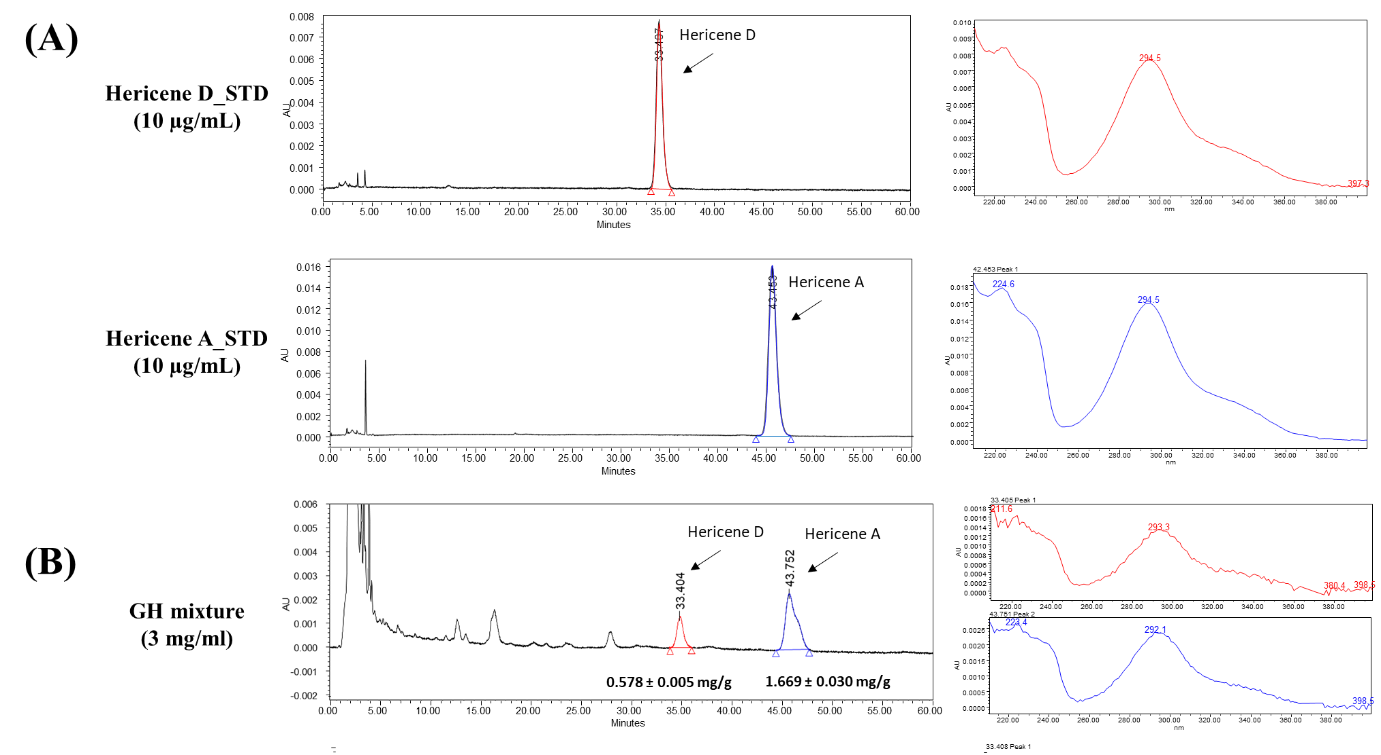


**Figure 5S. Chromatograms of standard compounds (ginkgolide A and ginkgolide D) and GH mixture extract (B).** Amounts of each compound was analyzed using HPLC with 72.5% water/17.5% water/10% isopropanol isocratic system at UV 340 nm.


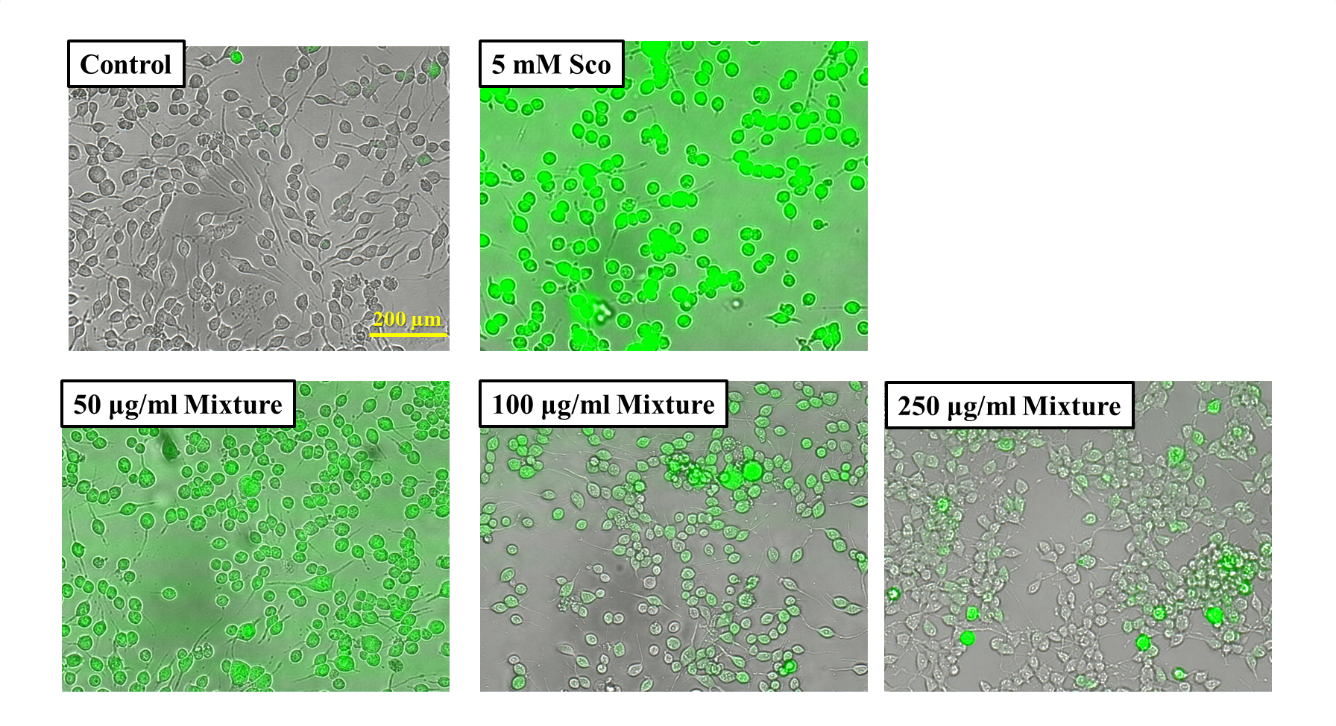


**Figure 6S. Effect of the mixture (GH) of *Ginkgo biloba L.* leaf (GL) and *Hericium erinaceus (Bull.) Pers.* fruit (HE) extracts on scopolamine (Sco)-induced ROS production.** ROS production was measured using the DCF-DA staining assay. Cells were treated with GH (dissolved in PBS) at 50, 100, and 250 μg/ml for 1 h, and then 5 mM Sco was co-treated for 1 h. Green fluorescence (ROS generation) from 2′,7′- DCF-DA was examined by JuLI live-cell imaging system. Scale bar indicates 500 μm.


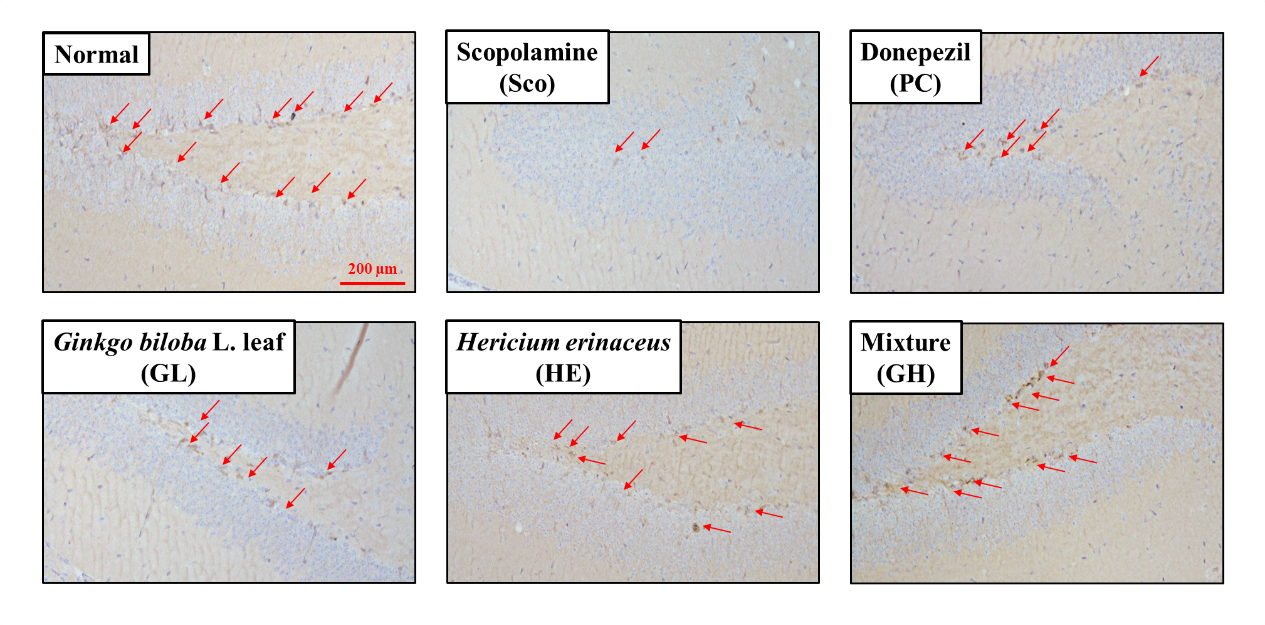


**Figure 7S. Effect of the mixture (GH) of *Ginkgo biloba L.* leaf (GL) and *Hericium erinaceus (Bull.) Pers.* fruit (HE) extracts on doublecortin (DCX) immunostaining assay.** The morphological changes of dentate gyrus (DG) region from hippocampus.
